# Pregnancy, Maternal Tobacco Smoking, and Early Age Leukemia in Brazil

**DOI:** 10.3389/fonc.2012.00151

**Published:** 2012-11-09

**Authors:** Jeniffer Dantas Ferreira, Arnaldo Cézar Couto, Maria S. Pombo-de-Oliveira, Sergio Koifman, Brazilian Collaborative Study Group of Infant Acute Leukemia

**Affiliations:** ^1^Environment and Public Health Post-graduation Program, National School of Public Health, Oswaldo Cruz FoundationRio de Janeiro, Brazil; ^2^Pediatric Hematology-Oncology Program, Research Center, Instituto Nacional de Câncer/Rio de JaneiroRio de Janeiro, Brazil; ^3^Members of the Brazilian Collaborative Study Group of Infant Acute Leukemia, listed in the appendix as co-authors

**Keywords:** smoking, pregnancy, childhood cancer, infant leukemia, lactation

## Abstract

**Background:** Cigarette smoking has been associated with acute myeloid leukemia (AML) but hypothesis on the association between maternal smoking during pregnancy and childhood leukemia remains unclear. **Objectives:** To investigate the association between maternal exposure to tobacco smoking during pregnancy and early age (<2 year) leukemia (EAL). **Methods:** A hospital-based multicenter case-control study aiming to explore EAL risk factors was carried out in Brazil during 1999–2007. Data were collected by direct interview with the biological mothers using a standardized questionnaire. The present study included 675 children (193 acute lymphoid leukemia – ALL, 59 AML and 423 controls), being the latter age frequency matched and paired by area of residence with the cases. Unconditional logistic regression was performed, and odds ratios (OR) on the association between tobacco smoking (3 months before pregnancy, during pregnancy, and 3 months after delivery) and EAL were ascertained after adjustment for selected variables (maternal age at birth and education, birth weight, infant skin color, and oral contraceptives use during pregnancy). **Results:** Smoking was reported by 17.5% of case mothers and 20.6% of controls. Among women who reported to have smoked 20 or more cigarettes during the index pregnancy, an adjusted OR = 5.28 (95% CI 1.40–19.95) for ALL was observed. Heavy smoking during breastfeeding yielded an adjusted risk estimate for ALL, OR = 7.78 (95% CI 1.33–45.5). No dose-response effect was observed according to smoking exposure during pregnancy and EAL. An association between secondhand smoking during pregnancy or breastfeeding was not observed. **Conclusion:** An association between maternal smoking and EAL in the offspring was restricted to women who have reported an intense exposure to tobacco smoke during pregnancy and breastfeeding.

## Introduction

The current smoking prevalence worldwide reaches around one billion persons (WHO, 2009). Epidemiologic research mainly conducted since 1950 has identified a causal association between tobacco smoking and lung cancer, as well as with 20 other tumor types (Secretan et al., 2009), chronic bronchitis, and ischemic heart diseases (Mackay and Eriksen, 2002).

In Brazil, data from a national population-based survey performed in 2008 indicated that 17.2% of individuals >14 year smoke, which represents about 25 million people (IBGE, 2008). Some detailed etiological mechanisms on the association between smoking and cancer remain unknown. Nevertheless, cigarette smoke spread over than 4,000 chemicals and toxic metals, including aluminum, ammonia, arsenic, benzene, DDT, dieldrin, carbon monoxide, carbon dioxide, chloroform, formaldehyde, hydrogen, lead, tar, and vinyl chloride. More than 40 of these chemicals are known carcinogens (Hecht, 1999; Chang et al., 2006; Thielen et al., 2008), 20 of them being probably involved in the induction of lung cancer as observed in animal studies (Hecht, 1999), and 11 considered human carcinogens (IARC, 2004). Moreover, tobacco smoke includes several chemicals which have been reported as leukemogenic in the literature, such as benzene (Korte et al., 2000; Hoffmann et al., 2001), pesticides (Rahman et al., 2012), arsenic (Yang, 2011), and metals (Yang, 2011).

According to leukemogenesis, the cytochrome-P450 enzymes family and NQO1 gene polymorphisms are involved in activation of benzene and other xenobiotic metabolites, both participating in benzoquinone detoxification and reactivating benzene intermediates (Zhang et al., 2010). Giving some support for an association between smoking and leukemia, a study carried out in Japan showed an increased risk for leukemia when occurring a joint occupational exposure to benzene in the presence of the NQO1 enzyme homozygous polymorphism [odds ratio, OR = 7.6 (95% CI 1.8–31.2; Rothman et al., 1997)].

Nicotine readily crosses the placenta and the fetal concentrations are 15% higher than maternal levels (Lambers and Clark, 1996). Nevertheless, maternal exposure to smoking during pregnancy was not associated with an increased risk of leukemia in a North-American study (Chang et al., 2006). However, paternal smoking during preconception showed statistically significant high risk estimates for acute myeloid leukemia (AML), OR = 3.84 (95% CI 1.04–14.17). When combined with maternal exposure during lactation, the study also found an increased ALL risk.

This study aimed to determine the magnitude of association between maternal exposure to smoking during preconception, pregnancy, and lactation, and the development of leukemia in children under 2 year in Brazil.

## Materials and Methods

### Study population

This investigation is part of a multicenter study named “Multi-institutional Study of Infant Leukemia: Contribution of Immunomolecular Markers in Distinguishing Different Etiopathogenic Factors,” which focuses on the investigation of the pathogenic mechanisms of early age leukemia (EAL) in Brazil (Pombo-de-Oliveira and Koifman, 2006). Participants (*n* = 675) were children <2 year, recruited from 15 different hospitals providing oncologic care, or general hospitals in all geographic areas in the country but the Amazon, including cities in the South, the Southeast, the Northeast, and the Midwest regions.

### Study design

This is a hospital-based multicenter case-control study in which controls were age frequency matched and paired by area of residence with enrolled cases. Data were obtained by interviews carried out with the respective patient mothers recruited in the Brazilian national universal healthcare system hospitals, which provide free care for pediatric patients either diagnosed with cancer or other illnesses.

### Case and controls ascertainment

Cases (*n* = 252) were defined as children ≤24 month old with a conclusive diagnosis of acute lymphoid leukemia (ALL) or AML, confirmed by morphologic, immunophenotypical, and cytogenetic-molecular standard methods. About 90% of ALL had confirmatory immunophenotyping, and both ALL and AML had been tested for specific lineage molecular diagnosis.

Controls (*n* = 423) were selected in the centers wherein cases were recruited, or general hospitals settled in the same cities, all belonging to the same age group and presenting non-malignant diseases. In order to reduce recall bias, they included hospitalized children with quite life-threatening conditions presenting the following distribution: infectious and parasitic diseases (*n* = 124, 29.4%); non-malignant hematological diseases (*n* = 83, 19.6%); asthma and bronchitis (*n* = 43, 10.2%); hemangiomas (*n* = 40, 9.4%); severe diarrhea (*n* = 39, 9.2%); cardiovascular diseases (*n* = 25, 5.8%); and others (*n* = 69, 16.4%).

### Exclusion criteria

Cases and controls with congenital syndromes, myelodysplasia, adoptive parents, or unknown biological mothers, were not included in this study. Controls with malignant or other benign tumor diagnosis were excluded.

### Data collection

The study was specifically designed to collect information on environmental exposures potentially associated with leukemogenesis. Data collection was gathered through face-to-face interview with case and control mothers, after signing a written informed consent. A standardized questionnaire containing the family’s socioeconomic background, parents’ occupational history, health antecedents, medicines use, as well as lifestyle patterns, including smoking, alcohol consumption, and other environmental exposures during pregnancy, was used.

Antecedents of reported maternal smoking were obtained, including smoking status at interview (never, past, and current smoking) and smoking during pregnancy. The latter included the usual amount of daily smoked cigarettes during preconception, pregnancy, and breastfeeding, and usual smoking frequency at these time windows: no primary hand smokers; moderate smokers (less than 20 smoked cigarettes per day); and heavy smokers (20 or more smoked cigarettes per day). Information on the lifelong length of tobacco exposure (in years), secondhand smoking, and smokers amount at home during pregnancy were also obtained.

### Ethical aspects

This study used primary data obtained from the project “Multi-institutional Study of Infant Leukemia: Contribution of Immunomolecular Markers in Distinguishing Different Etiopathogenic Factors.” This investigation was approved by the Research Ethics Committee of the Brazilian National Cancer Institute, No. 005/06.

### Study power

The initial sampling ascertainment forecast a sample size of 576 individuals, according to 2:1 controls per case ratio, totaling 192 cases and 384 controls for the study population. With a power of 80%, a type I error α = 5%, the size of the study enabled to detect minimum OR of 1.8 for an exposure prevalence among controls of about 30%.

### Statistical analysis

Unconditional logistic regression was performed to estimate the magnitude of association between maternal smoking and EAL, being the respective OR and their 95% confidence intervals ascertained after adjustment for selected variables (maternal age and education, oral contraceptives use during pregnancy, birth weight, and skin color) previously identified as confounders in the studied dataset (Pombo-de-Oliveira and Koifman, 2006). Considering the inclusion of some controls with asthma and cardiovascular diseases, a sensitivity analysis was performed to evaluate the overall impact of these participants in the ascertained association measures.

## Results

The main socio-demographic participant characteristics reveal statistically significant differences between cases and controls regarding maternal age, skin color, education, and income (Table 1). Thus, approximately 67% of cases were white (36% of controls), 60% of case mothers were 25 year or older (43% among controls), and 58% of the former reported school attendance of 8 years or more (49% among controls).

**Table 1:** **Distribution of selected maternal and child socio-demographic variables, leukemia cases, and controls, children <2 year, Brazil, 1999–2007**.

	Cases(*n* = 252), *n* (%)	Controls(*n* = 423), *n* (%)	*p*-Value
**SEX**			
Male	130 (51.6)	226 (53.4)	0.643
Female	122 (48.4)	197 (46.6)	
**BIRTH WEIGHT (g)**			
<4000	234 (92.8)	393 (93.0)	0.470
>4000	16 (6.2)	21 (5.0)	
**SKIN COLOR**			
White	170 (67.5)	153 (36.2)	<0.01
Non-White	77 (30.5)	256 (60.5)	
**PLACE OF BIRTH**			
Northeast	52 (20.6)	101 (24.1)	0.552
Midwest	18 (7.1)	31 (7.3)	
Southeast	155 (61.5)	237 (56.0)	
South	27 (10.7)	53 (12.5)	
**MATERNAL AGE AT BIRTH^a^ (YEARS)**			
<18	8 (3.2)	60 (14.1)	<0.01
18–24	91 (36.1)	182 (42.9)	
25–34	117 (46.4)	145 (34.2)	
>35	36 (14.3)	36 (8.8)	
**MATERNAL EDUCATION (YEARS)**			
<8	81 (32.1)	206 (48.6)	<0.01
>8	146 (57.9)	209 (49.4)	

*^a^Maternal age at delivery*.

Maternal tobacco smoking during pregnancy was reported by 17.5% of case mothers and 20.6% of controls’. Among children 0–11 months, maternal smoking during the index pregnancy was reported by 18.2% of ALL mothers, 17.9% of AML, and 23.1% of control’s. According to children 11–23 months, these proportions were, respectively, 20.0, 6.5, and 16.7%. Heavy smoking (20 or more cigarettes daily) during the studied time windows of interest was reported by nine (4.71%) of ALL mothers and four (0.9%) of controls’ (Table 3).

No association between maternal smoking and ALL or AML was observed according to the child age strata, 0–11 and 12–23 months (Table 2). Considering the amount of daily smoked cigarettes, a statistically significant association was observed between maternal daily consumption of 20 or more cigarettes and ALL, adjusted (adj.) OR = 5.28, 95% CI 1.40–19.95 (Table 3). Nevertheless, no dose-response effect on the amount of smoked tobacco and EAL was verified.

**Table 2:** **Maternal smoking during pregnancy according to offspring age strata, leukemia cases, and control mothers, children <2 year, Brazil, 1999–2007**.

Tobacco smoking at pregnancy/child age	Controls (*n* = 423), *n* (%)	ALL (*n* = 193), *n* (%)	AML (*n* = 59), *n* (%)	ALL		AML	
				OR (95% CI)	OR Adjusted^a^ (95% CI)	OR (95% CI)	OR Adjusted^a^ (95% CI)
**MATERNAL SMOKING**							
0–11 Months							
No	196 (76.9)	72 (81.8)	23 (82.1)	1.00	1.00	1.00	1.00
Yes	59 (23.1)	16 (18.2)	5 (17.9)	0.74 (0.40–1.37)	0.65 (0.31–1.38)	0.72 (0.26–1.98)	0.41 (0.11–1.59)
12–23 Months							
No	140 (83.3)	84 (80.0)	29 (93.5)	1.00	1.00	1.00	1.00
Yes	28 (16.7)	21 (20.0)	2 (6.5)	1.25 (0.67–2.34)	1.38 (0.65–2.93)	0.35 (0.08–1.53)	0.46 (0.10–2.24)
**TIME WINDOW**							
**Preconception^b^**							
0–11 Months							
No	176 (69.0)	59 (67.0)	20 (71.4)	1.00	1.00	1.00	1.00
Yes	79 (31.0)	29 (33.0)	8 (28.6)	0.89 (0.38–2.11)	1.23 (0.68–2.22)	1.10 (0.65–1.84)	0.82 (0.30–2.25)
12–23 Months							
No	128 (76.2)	79 (75.2)	27 (87.1)	1.00	1.00	1.00	1.00
Yes	40 (23.8)	26 (24.8)	4 (12.9)	1.05 (0.60–1.86)	0.98 (0.50–1.92)	0.47 (0.16–1.44)	0.42 (0.13–1.39)
**First trimester**							
0–11 Months							
No	209 (82.0)	71 (80.7)	24 (85.7)	1.00	1.00	1.00	1.00
Yes	46 (18.0)	17 (19.3)	4 (14.3)	1.09 (0.59–2.02)	1.03 (0.49–2.18)	0.76 (0.25–2.29)	0.62 (0.16–2.34)
12–23 Months							
No	145 (86.3)	84 (80.0)	29 (93.5)	1.00	1.00	1.00	1.00
Yes	23 (13.7)	21 (20.0)	2 (6.5)	1.58 (0.82–3.02)	1.61 (0.74–3.51)	0.44 (0.10–1.95)	0.41 (0.08–2.09)
**Second trimester**							
0–11 Months							
No	210 (82.4)	76 (86.4)	25 (89.3)	1.00	1.00	1.00	1.00
Yes	45 (17.6)	12 (13.6)	3 (10.7)	0.74 (0.37–1.47)	0.93 (0.43–2.02)	0.56 (0.16–1.94)	0.38 (0.08–1.83)
12–23 Months							
No	146 (86.9)	89 (84.8)	29 (93.5)	1.00	1.00	1.00	1.00
Yes	22 (13.1)	16 (15.2)	2 (6.5)	1.19 (0.60–2.39)	1.37 (0.61–3.07)	0.46 (0.10–2.05)	0.44 (0.09–2.29)
**Third trimester**							
0–11 Months							
No	211 (82.7)	76 (86.4)	25 (89.3)	1.00	1.00	1.00	1.00
Yes	44 (17.3)	12 (13.6)	3 (10.7)	0.76 (0.38–1.51)	0.97 (0.45–2.11)	0.58 (0.17–1.99)	0.40 (0.08–1.91)
12–23 Months							
No	146 (86.9)	89 (84.8)	29 (93.5)	1.00	1.00	1.00	1.00
Yes	22 (13.1)	16 (15.2)	2 (6.5)	1.19 (0.60–2.39)	1.50 (0.66–3.41)	0.46 (0.10–2.05)	0.49 (0.09–2.57)
**Breastfeeding^c^**							
0–11 Months							
No	209 (82.0)	77 (87.5)	26 (92.9)	1.00	1.00	1.00	1.00
Yes	46 (18.0)	11 (12.5)	2 (7.1)	0.65 (0.32–1.32)	0.90 (0.42–1.96)	0.35 (0.08–1.53)	0.39 (0.08–1.89)
12–23 Months							
No	148 (88.1)	88 (83.8)	29 (93.5)	1.00	1.00	1.00	1.00
Yes	20 (11.9)	17 (16.2)	2 (6.5)	1.43 (0.71–2.87)	1.86 (0.83–4.20)	0.51 (0.11–2.03)	0.69 (0.14–3.53)

*^a^Adjusted OR by maternal age at birth, maternal education, oral contraceptives use during pregnancy, birth weight, and infant skin color*.

*^b^Three months before pregnancy*.

*^c^Three months after birth*.

**Table 3:** **Secondhand maternal smoking, cigarettes consumption amount and duration of tobacco exposure, leukemia cases, and control mothers, children <2 year, Brazil, 1999–2007**.

Smoking	Controls (*n* = 423), *n* (%)	ALL (*n* = 193), *n* (%)	AML (*n* = 59), *n* (%)	ALL		AML	
				OR (95% CI)	OR Adjusted^a^ (95% CI)	OR (95% CI)	OR Adjusted^a^ (95% CI)
**SECONDHAND SMOKING**							
**Non-smoker mothers^b^**							
No	168 (51.9)	81 (56.6)	32 (66.7)	1.00	1.00	1.00	1.00
Yes	156 (48.1)	62 (43.4)	16 (33.3)	0.82 (0.56–1.23)	0.96 (0.63–1.47)	0.54 (0.28–1.02)	0.66 (0.34–1.29)
**FORMER SMOKER MOTHERS^c^**							
No	34 (38.6)	13 (24.1)	8 (50.0)	1.00	1.00	1.00	1.00
Yes	54 (61.4)	41 (75.9)	8 (50.0)	1.99 (0.93–4.23)	1.88 (0.81–4.34)	0.63 (0.22–1.84)	0.78 (0.22–2.75)
**SMOKER MOTHERS^d^**							
No	15 (18.3)	4 (14.7)	2 (33.3)	1.00	1.00	1.00	1.00
Yes	67 (81.7)	29 (85.3)	4 (66.7)	1.29 (0.43–3.91)	1.62 (0.43–6.14)	0.45 (0.08–2.68)	0.67 (0.07–6.10)
**EXPOSURE AMOUNT DURING PREGNANCY^e^ (CIGARETTES PER)**							
No use	304 (71.9)	137 (71.0)	48 (81.4)	1.00	1.00	1.00	1.00
1–5	62 (14.7)	34 (17.6)	7 (11.9)	1.22 (0.77–1.94)	1.18 (0.70–2.02)	0.72 (0.31–1.65)	0.69 (0.27–1.74)
6–19	53 (12.5)	13 (6.7)	4 (6.8)	0.54 (0.29–1.03)	0.61 (0.30–1.24)	0.48 (0.17–1.38)	0.37 (0.11–1.28)
20 or more	4 (0.9)	9 (4.7)	0 (0.00)	4.99 (1.51–16.5)	5.28 (1.40–19.9)	0.00	0.00
	*p*-trend = 0.75					
**EXPOSURE LENGTH (YEARS)**							
0–5	18 (24.0)	3 (12.5)	1 (25.0)	1.00	1.00	1.00	1.00
6–9	21 (28.0)	7 (29.2)	1 (25.0)	2.00 (0.45–8.89)	1.43 (0.29–7.06)	0.86 (0.05–14.71)	0.51 (0.02–11.15)
10–14	16 (21.3)	6 (25.0)	1 (25.0)	2.25 (0.48–10.50)	1.24 (0.21–7.43)	1.13 (0.07–19.50)	1.00 (0.04–21.74)
15+	20 (26.7)	8 (33.3)	1 (25.0)	2.40 (0.55–10.46)	1.74 (0.29–10.32)	0.90 (0.05–15.47)	0.22 (0.00–15.52)
	*p*-trend = 0.32					

*^a^Adjusted OR by maternal age at birth, maternal education, oral contraceptives use during pregnancy, birth weight, and infant skin color*.

*^b^Non-smoker mothers living with smokers*.

*^c^Former smoker mothers living with smokers*.

*^d^Smoker mothers living with smokers*.

*^e^Mean daily maternal cigarettes consumption during pregnancy*.

Enrolled children with a diagnosis of asthma (*n* = 18) and cardiovascular diseases (*n* = 28) accounted for 10.8% of controls. A sensitivity analysis was performed excluding them, and small changes on the risk estimates were observed. For instance, the magnitude of association between maternal heavy smoking during the third trimester (Table 4) changed from adj. OR = 7.78, 95 CI 1.33–45.5 (all controls included) to adj. OR = 6.98, 95% CI 1.21–40.4 (asthma and cardiovascular diseases excluded). A similar pattern was observed for other time windows of exposure (data not shown).

**Table 4:** **Maternal smoking by tobacco consumption burden, leukemia cases, and control mothers, children <2 year, Brazil, 1999–2007**.

Timing of exposure/smoking burden	Controls (*n* = 423), *n* (%)	ALL (*n* = 193), *n* (%)	AML (*n* = 59), *n* (%)	ALL		AML	
				OR (95% CI)	OR Adjusted^a^ (95% CI)	OR (95% CI)	OR Adjusted^a^ (95% CI)
**PRECONCEPTION^b^**							
Non-smokers	304 (71.9)	137 (71.0)	47 (79.7)	1.00	1.00	1.00	1.00
Moderate smokers	109 (25.7)	45 (23.3)	11 (18.6)	0.92 (0.61–1.37)	0.93 (0.59–1.45)	0.65 (0.33–1.30)	0.59 (0.27–1.28)
Heavy smokers	10 (2.4)	11 (5.7)	1 (1.7)	2.44 (1.01–5.88)	2.52 (0.95–6.72)	0.65 (0.81–5.17)	0.80 (0.93–6.90)
**FIRST TRIMESTER**							
Non-smokers	354 (83.7)	154 (79.8)	53 (89.8)	1.00	1.00	1.00	1.00
Moderate smokers	65 (15.4)	33 (17.1)	5 (8.5)	1.17 (0.74–1.85)	1.10 (0.65–1.82)	0.51 (0.20–1.33)	0.44 (0.15–1.31)
Heavy smokers	4 (0.9)	6 (3.1)	1 (1.7)	3.45 (0.96–12.39)	3.35 (0.75–15.0)	1.67 (0.18–15.23)	2.70 (0.27–26.5)
**SECOND TRIMESTER**							
Non-smokers	356 (84.2)	164 (85.0)	54 (91.5)	1.00	1.00	1.00	1.00
Moderate smokers	65 (15.4)	23 (11.9)	4 (6.8)	0.77 (0.46–1.28)	0.47 (0.02–0.92)	0.41 (0.14–1.16)	0.17 (0.01–2,14)
Heavy smokers	2 (0.4)	6 (3.1)	1 (1.7)	6.51 (1.30–32.0)	6.82 (1.09–4.30)	3.30 (0.30–37.0)	5.97 (0.47–76.0)
**THIRD TRIMESTER**							
Non-smokers	357 (84.4)	165 (85.5)	54 (91.5)	1.00	1.00	1.00	1.00
Moderate smokers	64 (15.2)	22 (11.4)	4 (6.8)	0.74 (0.44–1.25)	0.96 (0.54–1.69)	0.41 (0.14–1.16)	0.43 (0.10–1.18)
Heavy smokers	2 (0.4)	6 (3.1)	1 (1.7)	6.49 (1.30–32.0)	6.81 (1.09–42.7)	3.30 (0.30–37.0)	6.03 (0.47–77.0)
**BREASTFEEDING^c^**							
No smokers	357 (84.4)	164 (85.0)	55 (93.2)	1.00	1.00	1.00	1.00
Moderate smokers	64 (15.2)	22 (11.4)	3 (5.1)	0.75 (0.45–1.26)	0.94 (0.53–1.66)	0.30 (0.09–1.00)	0.37 (0.11–1.28)
Heavy smokers	2 (0.4)	7 (3.6)	1 (1.7)	7.62 (1.57–32.0)	7.78 (1.33–45.5)	3.25 (0.29–36.4)	6.05 (0.47–77.0)

*^a^Adjusted OR by maternal age at birth, maternal education, oral contraceptives use during pregnancy, birth weight, and infant skin color*.

*^b^Three months before pregnancy*.

*^c^Three months after birth*.

Not statistically significant increased EAL risk estimates associated with a cumulative exposure to tobacco smoking were observed for ALL in all quartiles of time length of tobacco exposure. Comparatively to the first quartile (0–5 years) used as reference, the following risk estimates were verified: adj. OR = 1.43, 95% CI 0.29–7.06 in the second smoking quartile (6–9 year); adj. OR = 1.24, 95% CI 0.21–7.43 in the third quartile (10–14 year); adj. OR = 1.74, 95% CI 0.29–10.3 in the fourth quartile [14 year or more, *p*-trend = 0.32 (Table 3)]. No association was also observed according to maternal secondhand smoking.

## Discussion

Although smoking has historically been a predominantly male-related behavior until the beginning of the twentieth century (Einarson and Riordan, 2009), women’s liberation movement during the 1960s and 1970s accelerated a lifestyle change, with smoking increase among women in industrialized countries. Hence, increasing rates of several smoking health related hazards among women started to be observed in Brazil and other countries (Lombardi et al., 2010). Such epidemiological change has stimulated the organization of public policies toward smoking control programs addressed to prevent such addiction among women (Amos et al., 2012).

Eleven cigarette compounds were detected as human carcinogens and others may be carcinogenic, but have not been yet fully evaluated (IARC, 2004). Tobacco smoke also contains tumor promoters (phenolic substances), co-carcinogens (catechol and related compounds), toxic agents (acrolein and other aldehydes), and free radical species (nitric oxide and others; IARC, 2004).

Even though, the role of parental prenatal smoking in childhood leukemia remains unknown. Nevertheless, such biological plausibility is supported by the presence of carcinogenic chemicals in tobacco smoke (IARC, 2004; Thielen et al., 2008), and their ability to cross the placenta (de la Chica et al., 2005), to cause DNA damage (Potts et al., 1999), oxidative damage (Fraga et al., 1996), chromosomal abnormalities (Pluth et al., 2000), and aneuploidy in human sperm (Shi et al., 2001).

Maternal tobacco smoking during pregnancy was reported in this study by 17.5% of case mothers and 20.6% of controls’. Data from a population-based national survey carried out in 2008 indicated that smoking prevalence in women 15 year or older was 13.1% in Brazil (IBGE, 2008). According to other national population-based survey with phone interviews, such figures are higher in the South and Southeast of Brazil, wherein they range around 22% among women, being lower in the Northeast, around 6% (Brazil, 2009).

The null association between maternal smoking and EAL risk regardless the period of exposure during pregnancy, as observed in this study, is consistent with the literature. The absence of an association between maternal smoking exposure and EAL according to the child age strata in this investigation, does not support an association between the occurrence of MLL gene rearrangements as a consequence of such exposure, considering the high prevalence of such rearrangements observed in infant leukemia (Marschalek, 2011).

On the other hand, an association between maternal smoking during pregnancy and EAL cannot be completely ruled out, since a statistically significant association was observed for heavy smokers and ALL, adj. OR = 5.28, 95% CI 1.40–19.95. Hence, this study results suggest that such association may occur, if the amount of tobacco daily exposure remains high during pregnancy or immediately later. Nevertheless, it is important to remark that these results were observed with few heavy smoker mothers (nine ALL and four control mothers). Additionally, the variable smoking consumption was highly correlated during the time windows of exposure (Pearson correlation coefficients ranging from *r* = 0.66 between preconception and breastfeeding, and *r* = 0.85 between the third trimester and breastfeeding). In regard to secondhand smoking during pregnancy, no association with EAL was observed.

A research published by IARC/WHO concluded that passive smoke is carcinogenic (IARC, 2004), and smoke inhaled by passive smokers is responsible for tobacco-related diseases, especially lung cancer (Hackshaw et al., 1997). Passive smoking may begin during the intrauterine life, due to pregnant smokers, workplaces and living with smokers, introducing toxic substances through the umbilical cord to the fetus. Besides differences according to carcinogenesis in early life, infants have distinct capacities from adults to metabolize and clear chemicals, which can result in larger or smaller internal doses of active agents, either increasing or decreasing risk (Ginsberg et al., 2002).

A case-control study conducted in Australia (Milne et al., 2012) examined the association between parental smoking and the risk of childhood ALL. Maternal smoking was not associated with risk of childhood ALL, but a performed meta-analyses suggested that heavier paternal smoking around the time of conception is a risk factor for childhood ALL (OR = 1.44, 95% CI 1.24–1.68).

A Canadian study observed an association between AML and 10–19 cigarettes per day consumption during pregnancy, OR = 3.89, 95% CI 1.31–11.58. The risk estimate for the same intake during the first trimester was OR = 4.03, 95% CI 1.33–12.21 (MacArthur et al., 2008). Conversely, two French studies did not find an association between maternal smoking and childhood leukemia. The first one did not either observed an association before, or during pregnancy, or during childhood (Menegaux et al., 2007). The other study did not detect an association regardless the maternal period of smoking during pregnancy (Menegaux et al., 2005). In a national registry-based case-control carried out in France (Rudant et al., 2008), an association between maternal smoking and childhood leukemia was also not observed. In this sense, only few studies have found maternal smoking to be significantly associated with the risk of ALL (Sorahan et al., 2001; MacArthur et al., 2008).

The analyzed data in the current investigation were strictly dependent on a maternal report, which may have introduced incorrect exposure estimates, being over or underreported. Nevertheless, we believe that whether this lack of accuracy has occurred, it probably was non-differential, considering the use of a standardized questionnaire and interview procedures to get information from case and control mothers, which decreases the potential differential misclassification. Thus, we consider that the observed results are not likely to be explained by underreporting smoking controls, since the proportion of mothers who reported to have smoked during the study was higher than the average percentage of Brazilian population in 2003 (18.4%) and 2008 (13.1%; Wunsch Filho et al., 2010). However, mothers of reported cases may have been not exposed to their own cigarettes, thus reducing the chance to produce an observable association. On the other hand, Menegaux et al. (2005) suggested that the general feeling of guilty associated with smoking may be stronger in mothers of children with leukemia than among control’s, arising unequal accuracy of information provided by the former.

Most of the literature reveals a relationship between the risk of childhood leukemia and paternal smoke around the time of conception, probably due to its influence in spermatogenesis (Shu et al., 1996; Pang et al., 2003; Chang et al., 2006; Rudant et al., 2008; Lee et al., 2009; Milne et al., 2012). In this investigation, paternal smoking data was not obtained. Therefore, evaluation whether the offspring of heavy smoker mothers – showing higher EAL risk estimates – also had smoker fathers, was unfeasible to be accomplished.

Maternal smoking during pregnancy has been associated to several health hazards, including spontaneous abortion, infertility, and sudden infant death syndrome, among several others (Einarson and Riordan, 2009). A higher incidence of spontaneous abortion among case mothers, comparatively to controls’, could yield to a null association between smoking and leukemia. If true, however, such fact would mainly target heavy smoker pregnants, and a null association would mainly occur in their offspring, which was not observed. Additionally, it is possible that heavy smoker mothers were less likely to be included in the current study as a consequence of the association between smoking with abortion and infertility.

In the current investigation, the choice of hospitalized controls with severe diseases may have probably mitigated the occurrence of such kind of bias. Controls were selected from the same regions wherein cases came from, as a procedure to assure that the study base principle could be accomplished, i.e., that all enrolled participants could share the background pattern of exposures, thus allowing to obtain unbiased risk estimates with the performed comparisons (Wacholder et al., 1992). Finally, according to AML, the relatively small amount of enrolled cases in this study did not allow the ascertainment of some risk estimates.

This investigation presented some weaknesses. At first, sample size of AML cases limited data exploratory analysis. Secondly, considering the high correlation among the reported smoking prevalence in the different time windows of exposure, the obtained risk estimates in each of them are probably imprecise, and they need to be interpreted cautiously. Thirdly, data on paternal smoking before and during pregnancy was not collected during the interviews.

Nevertheless, the study presents some strengths. At first, it was feasible to collect maternal data exposure to smoking in a very rare cancer, such as leukemia in children under 2 year. At such age strata, with a short postnatal life span of exposures, the importance of *in utero* environmental exposures is of paramount importance, and the investigation was able to either obtain a detailed report about timing and amount of tobacco (smoked cigarettes) exposure during pregnancy, or other important risk factors, such as birth weight and oral contraceptives use during the same period. Finally, to our knowledge, this is the first Latin American study exploring the association between maternal smoking during pregnancy and leukemia among children under 2 year.

Consistent with most previous studies (Menegaux et al., 2005, 2007), this study did not report an association between maternal smoking and EAL. This result is in agreement with a possible lower maternal contribution to the risk of embryonic genetic mutations, comparatively to paternal one. Such observation can result to the fact that the ova are formed and stored in the female embryo, being better protected against genotoxic stress, while the male germ cells, keeps the process continuously (Guerquin et al., 2009). However, maternal daily consumption of 20 or more cigarettes during pregnancy showed a fivefold higher statistically significant association with the risk of ALL. Such risk estimates are indeed higher if considered such exposure during the second trimester of pregnancy, the third trimester, or breastfeeding.

To conclude, this investigation results observed the occurrence of an association between an intensive exposure to tobacco smoking during pregnancy and AAL, being higher if such exposure occurred after the first trimester of pregnancy, including breastfeeding.

## Conflict of Interest Statement

The authors declare that the research was conducted in the absence of any commercial or financial relationships that could be construed as a potential conflict of interest.

## Appendix

List of pediatricians and researchers from the *Brazilian Collaborative Study Group of Infant Acute Leukemia* who have contributed as co-authors to the study.

Jane Dobbin^1^, Jozina Maria de Andrade Agareno^2^, Alejandro Aranciba^3^, Flávia Nogueira Serafim Araújo^2^, Rosania Baseggio^4^, Reinaldo Del Belo^1^, Silvia Brandalise^5^, Lilian M Burlacchini de Carvalho^6^, Eni Guimarães de Carvalho^6^, Tereza Cristina Cardoso^6^, Imaruí Costa^7^, Jose Carlos Cordoba^8^, Virginia M Coser^9^, Maria Lucia Lee^10^, Renato Melarangno^3^, Núbia Mendonça^2^, Isis Q Magalhães^8^, Atalla Mnayarji^4^, Cynthia Curvello Neves^2^, Flávia Pimenta^11^, Mara A.D.Pianovsky^12^, Vitória Pinheiro^5^, Terezinha JM Salles^13^, Fernando Werneck^14^ and César Bariani^15^

^1^ Research Center and Hematology Service, Instituto Nacional de Câncer, Rio de Janeiro, Rio de Janeiro, Brazil

^2^ Pediatric Hematology-Oncology Service, Hosiptal Santa Izabel, Salvador, Bahia, Brazil

^3^ Pediatric Hematology-Oncology Service, Hospital Santa Marcelina, São Paulo, São Paulo, Brazil

^4^ Pediatric Hematology-Oncology Service, Hospital Rosa Pedrossian, Campo Grande, Mato Grosso do Sul, Brazil

^5^ Centro Infantil de Investigações Hematológicas D. Boldrini, Campinas, São Paulo, Brazil

^6^ Pediatric Hematology-Oncology Service, Hospital Martagão Gesteira, Salvador, Bahia, Brazil

^7^ Pediatric Hematology-Oncology Service, Hospital Joana de Gusmão, Florianópolis, Santa Catarina, Brazil

^8^ Hospital de Apoio Brasília, Unidade de Onco-Hematologia Pediátrica, Brasília, Federal District, Brazil

^9^ Departamento de Hematologia, Universidade de Santa Maria, Santa Maria, Rio Grande do Sul, Brazil

^10^ Pediatric Oncology Institute- GRAAC, São Paulo, São Paulo, Brazil

^11^ Hospital Napoleão Laureano, João Pessoa, Paraíba, Brazil

^12^ Hospital Pegueno Principe, Curitiba, Paraná, Brazil

^13^ Hospital Oswaldo Cruz, Recife, Pernambuco, Brazil

^14^ Pediatric Oncology Section, Hospital dos Servidores do Estado do Rio de Janeiro, Rio de Janeiro, Rio de Janeiro, Brazil

^15^Serviço de Transplantes de Medula do Hospital Araújo Jorge, Goiania, Goiás, Brazil
